# Isothermal DNA amplification coupled to Au-nanoprobes for detection of mutations associated to Rifampicin resistance in Mycobacterium tuberculosis

**DOI:** 10.1186/1477-3155-11-38

**Published:** 2013-11-25

**Authors:** Bruno Veigas, Pedro Pedrosa, Isabel Couto, Miguel Viveiros, Pedro V Baptista

**Affiliations:** 1 Nanotheranostics@CIGMH, Departamento de Ciências da Vida, Faculdade de Ciências e Tecnologia, Universidade Nova de Lisboa, Campus de Caparica 2829-516, Caparica, Portugal; 2 CENIMAT/I3N, Departamento de Ciência dos Materiais, Faculdade de Ciências e Tecnologia, Universidade Nova de Lisboa, Caparica, Portugal; 3 Grupo de Micobactérias, Unidade Microbiologia Médica, Universidade Nova de Lisboa (IHMT/UNL), Lisboa, Portugal; 4 CREM, Centro de Recursos Microbiológicos, Universidade Nova de Lisboa, Lisboa, Portugal; 5 Centro de Malária e Outras Doenças Tropicais, Instituto de Higiene e Medicina Tropical, Universidade Nova de Lisboa (IHMT/UNL), Lisboa, Portugal

**Keywords:** MDRTB, Nanodiagnostics, LAMP, PCR, Gold nanoparticles, Tuberculosis, DNA isothermal amplification, Rifampicin

## Abstract

**Background:**

Tuberculosis accounted for 8.7 million new cases in 2011 and continues to be one of the leading human infectious diseases. Burdensome is the increasing rate of multi-drug resistant tuberculosis (MDRTB) and the difficulties created for treatment and public health control programs, especially in developing countries. Resistance to rifampicin (RIF), a first line antibiotic, is commonly associated with point mutations within the *rpoB* gene of *Mycobacterium tuberculosis* (Mtb) whose detection is considered the best early molecular predictor for MDRTB. Gold nanoparticles functionalized with thiol-modified oligonucleotides (Au-nanoprobes) have shown the potential to provide a rapid and sensitive detection method for Mtb and single base alterations associated with antibiotic resistance, namely in *rpoB* gene associated to RIF resistance.

**Results:**

We developed a strategy based on the isothermal amplification of sample DNA (LAMP) coupled to specific Au-nanoprobes capable of identifying members of the Mtb complex (MTBC) and discriminating specific mutations within the *rpoB* gene. Integration of LAMP and Au-nanoprobe assay allowed to detect MTBC member and identify mutations linked to RIF resistance. A total of 12 biological samples were tested and a 100% specificity and sensitivity was attained.

**Conclusions:**

There is an increasing demand for simple, fast and cheap methods for the molecular identification of Mtb and for the detection of molecular tags associated to drug resistance suitable for use at point-of-need. Here we describe such a method, that as the potential to get molecular diagnostic of tuberculosis to remote environments.

## Background

Tuberculosis (TB) is still one of the leading human infectious diseases with reports of 8.7 million new cases in 2011 [1]. Amongst these, of particular concern is the increasing rate of antibiotic resistance, namely for first line antibiotics, such as Rifampicin (RIF) [1]. The emergence of drug resistant *M. tuberculosis* poses a serious threat to the TB control programs and the TB cure rates since patients infected with drug resistance strains are difficult to treat and are likely to remain as sources of infection for longer periods of time. Resistance to RIF has been associated to single point alterations within a well-defined 81 bp region (codons 507–533) of the *rpoB* gene encoding for the beta subunit of RNA polymerase [2,3]. More than 35 distinct single base mutations have been described within this region conferring resistance to RIF and among these, two mutations - H526D and S531L - account for two-thirds of RIF resistance and are absent in susceptible isolates, making them ideal molecular tags for detection strategies [2-4]. Access to point-of-need diagnostics in TB is the greatest obstacle for the early detection and identification of patients harboring resistant strains, since standard methods of diagnostic are either, cumbersome and expensive, or only exist at centralized laboratories. Most proposed systems thus far rely on the PCR amplification of the sample before detection [3,5]. Therefore, the development of cheap, fast and simple molecular methods to assess susceptibility profiles at point-of-care would have a huge impact in the capacity of early diagnosis and treatment of TB patients.

We have previously reported on a colorimetric non-cross-linking approach using gold nanoparticles (AuNPs) functionalized with thiol-modified DNA (Au-nanoprobes) for the detection of MTBC and characterization of mutations [6-8]. This approach is based on the different aggregation profile of Au-nanoprobes in presence/absence of the specific complementary target upon salt induced nanoparticle aggregation. Presence of the complementary target sequence to that of the probe prevents aggregation and the solution remains red (localized surface plasmon resonance (LSPR) band at 525 nm), whereas absence of a specific target sequence leads to extensive aggregation after salt addition and the solution turns blue (red-shift of the LSPR peak). The system is extremely sensitive and allows detection of the pathogen’s DNA directly from biological samples [9]. However, for robustness and detection of single base alterations, a PCR amplification step is usually required.

**Figure 1:**
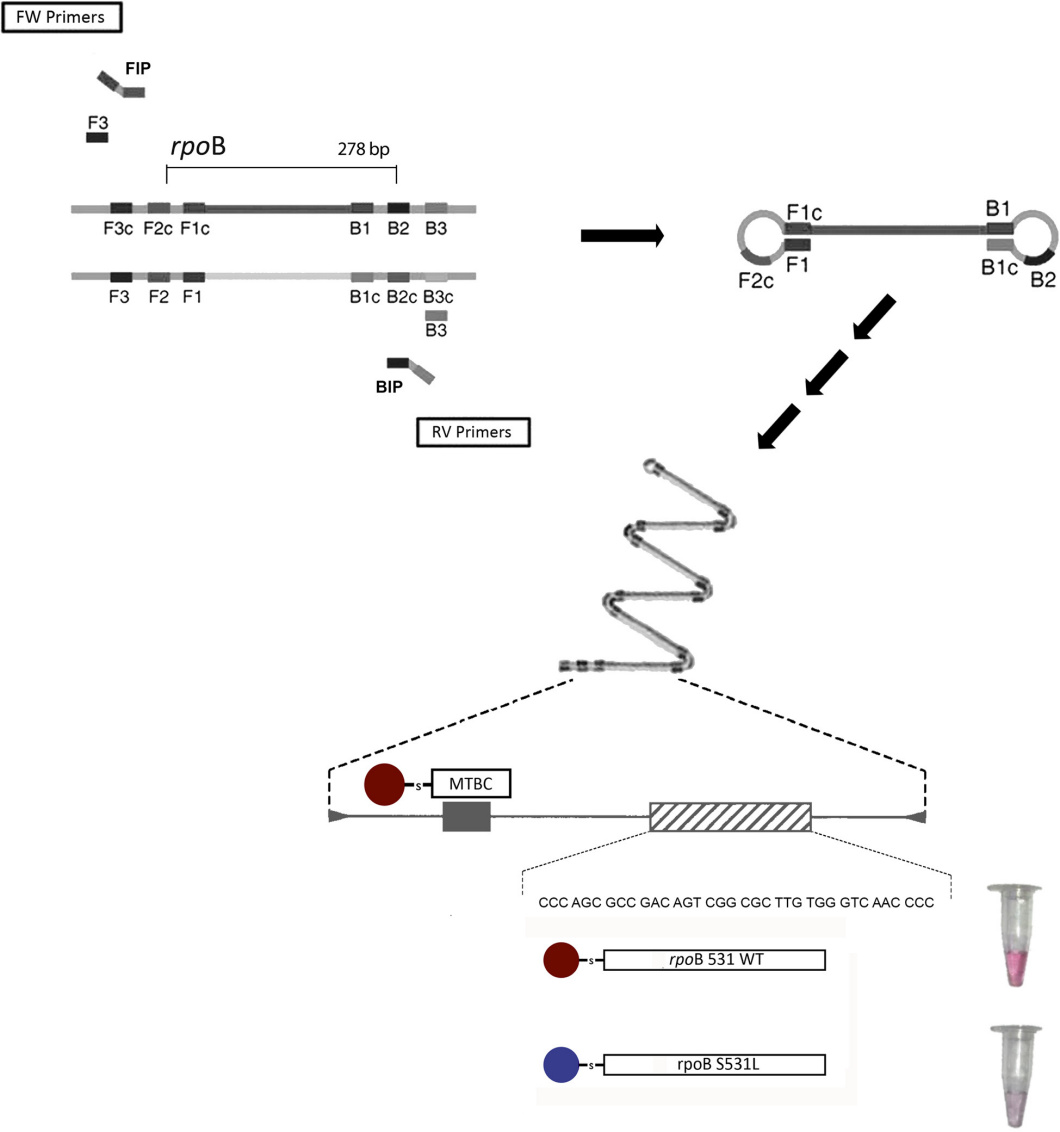
**Au-nanoprobe strategy for detection of MTBC members and mutations associated with RIF resistance.** Illustration of the LAMP amplification using primers specific for MTBC members and schematic representation of the *rpoB* gene and positions of primers and probes. Au-nanoprobe detection strategy of LAMP products (MTBC probe) and *rpoB* S531L mutation: The black box indicates the sequence recognized by the MTBC-probe. The dashed box with corresponds to the sequence recognized by the mutation specific probes derived from the *rpoB* gene. The colorimetric assay consists of visual comparison of test solutions before and after salt induced Au-nanoprobe aggregation.

Here, we overcome the need for PCR by coupling the Au-nanoprobe detection protocol to Loop-mediated isothermal amplification (LAMP) – see Figure 1. The use of LAMP for sample DNA amplification seems to be ideal for field analysis as its isothermal profile, increased specificity and speed, make this approach desirable over standard PCR, especially for point-of-need application [10]. Taking advantage of such features, we demonstrate that the non-cross-linking system is capable to discriminate the *rpoB* S531L point mutation on LAMP products and, thus, opening new possibilities for MDRTB diagnostics in remote environments and at a point-of-care.

## Methods

All reagents were purchased from Sigma Aldrich and were of analytical grade. HPLC purified labeled oligonucleotides were purchased from STABVida (Portugal) and used without further purification. Thiolated oligonucleotides were used to synthesize the Au-nanoprobes and non-modified oligonucleotides were used as controls for calibration of the assay.

Twenty-five clinical isolates obtained from respiratory samples positive for acid fast bacilli from patients of the Lisbon Health Region, including 6 strains susceptible and 6 resistant to RIF, were used. Additionally, one strain previously determined as Mtb H37Rv (ATCC27294^T^) was used as positive control and a non-MTBC strain (*M. kansasii*) as negative control. The BACTEC^TM^ MGIT^TM^ 960 (BACTEC 960) system was used for primary isolation and standard susceptibility testing for the first line drugs (streptomycin, isoniazid, RIF and ethambutol) according to the manufacturer’s instructions (Becton Dickinson Diagnostic Systems, Sparks, MD, USA). Identification of MTBC and mutations in the *rpo*B gene associated to RIF resistance was performed by INNO-LiPA Rif. TB assay (Innogenetics, Belgium). DNA was extracted from cultures with the QIAamp DNA Mini kit (QIAGEN, Hilden, Germany) according to the manufacturer’s instructions.

LAMP amplification and primer design were performed according to Notomi *et al*. [10] (see Table 1). Reactions were performed in a final volume of 50 μl including 1 μM of each inner primer FIP and BIP, 0.1 μM of each outer primer FP and BP, 0.3 mM of dNTP mix, 1× of the supplied buffer, 0.5 M betaine (Sigma–Aldrich, MO, USA), 2 mM MgCl_2_, and 10 ng of DNA. An initial step of 10 min denaturation at 95°C on a Bio-Rad MyCycler Thermocycler (BioRad, CA, USA) was performed and afterwards the mix was cooled down at 4°C for one minute. Following addition of 1U of *Bst* DNA polymerase, large fragment (New England Biolabs Inc., MA, USA), the reaction mixture was incubated for 30 min at 65°C. For the negative control, the same procedure was followed but sample DNA was substituted by water. Following LAMP, all samples were ethanol precipitated and centrifuged for 15 min at 14000 g, the pellet dried under vacuum and resuspended in sterile deionized water.

**Table 1:** LAMP primers and Au-nanoprobes sequences

**Name**	**Sequence**
*rpoB* BIP	5’ CCG GCG GTC TGT CAC GTG AAG TGC GAC GGG TGC A 3’
*rpoB* FIP	5’ TGG GTG GTC ATC CGC TCC CCA GAT CCG GGT CGG CAT G 3’
*rpoB* F3	5’ TCG GCG AGC TGA TCC AA 3’
*rpoB* B3	5’ CCC CTC AGG GGT TTC GA 3’
MTBC	Thiol - 5’ GAT CGC CTC CAC GTC C 3’
*rpoB* 531 WT	Thiol - 5’ GCC GAC AGT CGG CGC TTG TG 3’
*rpoB* 531 Mut	Thiol - 5’ GCC GAC AGT CGG CGC TTG TC 3’

Au-nanoprobes sequences were designed using Serial Cloner v. 1.3-11 and BioEdit 7.1.3.0 comparative tools aligning the probe sequence with the target *M. tuberculosis* gene. The Au-nanoprobe set for mutation detection consisted of two probes (see Table 1): one complementary to the wild type (WT) sequence and the other complementary to the mutated (Mut) sequence. AuNPs, with an average diameter of ~14 nm, were synthesized (see Additional file 1: Figure S1) and functionalized as described by Veigas *et al*. [7]. Briefly, AuNPs (15 nM) were mixed with thiol-modified oligonucleotides in a theoretical ratio of 1:200 AuNPs:oligo, thus increasing salt solutions were added in 20 min intervals to attain a final NaCl concentration of 0.3 M in order to reduce non-specific bonds between the thiol-modified oligonucleotides and the AuNPs. The solution was incubated during 16 hours and washed with 10 mM phosphate buffer (pH 8). The solution was centrifuged, the resulting pellet resuspended in 10 mM phosphate buffer (pH 8), 0.1 M NaCl, and stored in the dark at 4°C till further use. The minimum amount of salt (MgCl_2_) required to induce aggregation of the Au-nanoprobe in absence of the complementary target was determined at 30 mM of salt for each synthesized probe (see Additional file 1: Figure S2).

The Au-nanoprobe colorimetric assays were performed in a final volume of 30 μl containing Au-nanoprobes at a final concentration of 2.5 nM in 10 mM phosphate buffer (pH 8) and the LAMP DNA product at a concentration of ~30 ng/μL. The mixture was heated up at 95°C for 10 min and then cooled down to 20°C for 10 min. For each probe, the assay consisted on the spectrophotometric comparison of a “Blank” (without DNA), 10 mM phosphate buffer (pH 8), 0.1 M NaCl; POSITIVE control containing a complementary control DNA to the Au-nanoprobe; a NEGATIVE control containing non-complementary DNA; and the samples. The pre-determined MgCl_2_ amount was added to each reaction and, after 30 min at room temperature for color development, the mixtures and the blank assayed by UV/visible spectroscopy in a microplate reader (Tecan Infinite M200). For calibration purposes, each set of Au-nanoprobes was tested against purified simplex PCR amplicons.

Aggregation profiles were analyzed in terms of the Abs_525nm_/Abs_600nm_ ratio (dispersed *vs*. aggregated species) for each Au-nanoprobe. A minimum of three independent assays were performed for each sample and a threshold of 1 was considered where values >1 indicates dispersed Au-nanoprobes, whereas a value <1 indicates aggregation [11]. For the MTBC Au-nanoprobe, this approach provides for indication of presence or absence of MTBC DNA in the sample, respectively. When using a set of Au-nanoprobes targeting the WT and Mut sequences, and to simplify analysis, the Abs_525nm_/Abs_600nm_ ratio for the WT probe was divided by the ratio of the Mut prob. Values >1 identify cases where the WT probe is more stable than Mut probe, and thus the sample presented the WT sequence; whereas values <1 indicate the presence of mutation. For every sample, a minimum of three independent tests were performed and the mean value used.

## Results and discussion

LAMP primers were designed to amplify one of the most relevant *loci* for characterization of mutations associated with RIF resistance – *rpoB*. A single cut restriction enzyme followed by agarose gel electrophoretic analysis was used to confirm amplification of the desired amplicon (see Additional file 1: Figure S3). Since LAMP amplification does not rely on temperature cycles, faster amplification than standard PCR protocol for the target sequence is attained [12]. Then, 12 samples previously characterized for molecular signatures of susceptibility were successfully amplified by LAMP.

**Figure 2:**
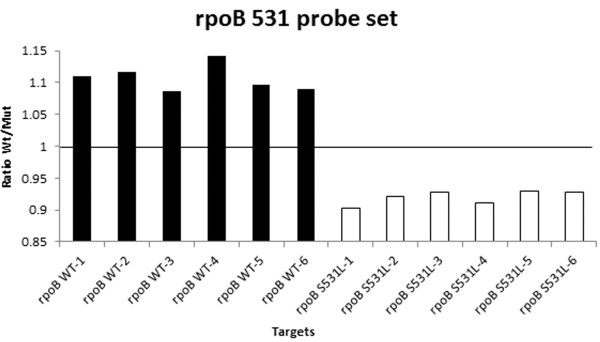
**LAMP products tested with *rpoB *probe set.** Au-nanoprobes detection assays of LAMP products, using the Abs_525nm_\Abs_600nm_ ratio of *rpoB* 531 WT probe divided by the ratio of *rpoB* Mut probe. Values higher than one are considered WT (Black) and lower than one Mut (White).

**Figure 3:**
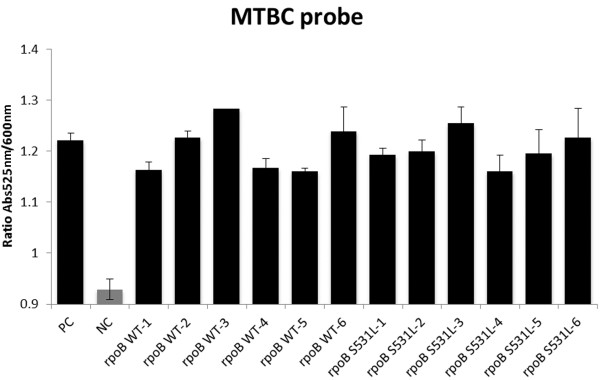
**LAMP products tested with MTBC probe.** PC - positive control; NC - negative control. A threshold of 1 was set to differentiate between positive (Black) and negative (Grey).

An Au-nanoprobe capable of identifying members of MTBC was synthesized and calibrated using synthetic oligos. LAMP products were then assayed with the MTBC Au-nanoprobe and all 12 samples were accurately scored as MTBC positive – see Figure 2. The additional Au-nanoprobe set, specific for one of the most common point mutations associated with RIF resistance (*rpoB* S531L), was also synthesized. This set is composed of two probes: one complementary to the WT sequence and the other complementary to the mutation, where each probe sequence was designed so as to be complementary to the target sequence with the possible mutation at the 3´ end of the Au-nanoprobe for better sequence discrimination [13]. All 12 samples amplified by LAMP were assessed with this Au-nanoprobe set, which was capable to correctly identify the genotype present with a specificity and sensitivity of 100%. Figure 3 shows that from 12 samples 6 were characterized as *rpoB* 531 WT and the other 6 as *rpoB* S531L (see also Additional file 1: Table S4).

We report on the development of an isothermal amplification coupled to Au-nanoprobe detection protocol for the simultaneous detection of members of MTBC and characterization of mutations associated with antibiotic resistance in *M. tuberculosis*. For the first time, it was possible to identify single point mutations on LAMP products via the use of Au-nanoprobes. Since LAMP originates long DNA concatemers, we demonstrate that Au-nanoprobes are capable to discriminate single base mismatches independently of DNA length and only dependent of differential hybridization WT and Mut Au-nanoprobes to the target DNA sequence.

We demonstrate that it is possible to use an Au-nanoprobe based strategy to detect single point alteration on isothermally amplified DNA products. Previous work of a colorimetric detection approach using Au-nanoprobes coupled to LAMP has been described [14-16], however this is the first time this approach is described for the identification of point mutations with biological samples. We further demonstrate that this method is highly specific for application to real biological samples. The LAMP-Au-nanoprobe strategy was capable to specifically identify MTBC members and mutations associated to RIF resistance in Mtb within 90 minutes of DNA extraction. Our technique has proven to be a fast and robust approach, ideal for the molecular diagnostic at point-of-need. The Au-nanoprobes strategy discards the use of electrophoretic analysis of LAMP products thus speeding the results and reducing costs; it also as the possibility to be extended, to analyze more mutation with a single LAMP. Future work will be focused on the optimization of this strategy towards assessment of other mutations associated with MDRTB.

## Abbreviations

AuNPs: Gold nanoparticles; LAMP: Loop-mediated isothermal amplification; LSPR: Localized surface plasmon resonance; MDRTB: Multi-drug resistant tuberculosis; Mtb: *Mycobacterium tuberculosis*; MTBC: *Mycobacterium tuberculosis* complex; Mut: Mutated; RIF: Rifampicin; TB: Tuberculosis; Wt: Wild type.

## Competing interests

The authors declare that they have no competing interests.

## Authors’ contributions

BV and PP participated equally in the development of the methodology, design of the LAMP amplification procedure, carried out the nanoprobe synthesis, performed the detection assays and drafted the manuscript. MV and IC participated in the design of the study and characterized the biological samples through standard diagnostic methodologies. PB conceived the study, participated in its design and coordination, and drafted the manuscript. All authors read and approved the final manuscript.

## Supplementary Material

Additional file 1 **Characterization of AuNPs, Au-nanoprobes and LAMP products.** Additional figures of AuNPs, Au-nanoprobes and LAMP products characterization: TEM analysis of AuNPs, with nanoparticle counts and TEM image; effect of salt in the stability of Au-nanoprobes; electrophoretic analysis of LAMP product. Click here for file
