# Supplementary material for: Isothermal DNA amplification coupled to Au-nanoprobes for detection of mutations associated to Rifampicin resistance in Mycobacterium tuberculosis
Source: J Nanobiotechnology. 2013 Nov 25;11:38. doi: 10.1186/1477-3155-11-38 (PMC4222569; doi:10.1186/1477-3155-11-38)
Supplement: Additional file 1 — Characterization of AuNPs, Au-nanoprobes and LAMP products. Additional figures of AuNPs, Au-nanoprobes and LAMP products characterization: TEM analysis of AuNPs, with nanoparticle counts and TEM image; effect of salt in the stability of Au-nanoprobes; electrophoretic analysis of LAMP product. [file 1477-3155-11-38-S1.pdf]

Journal of Nanobiotechnology

Supplementary Data

LAMP coupled to Au-nanoprobes for detection of mutations associated to Rifampicin resistance in *Mycobacterium tuberculosis*

Bruno Veigas[1,2,†], Pedro Pedrosa[1,†], Isabel Couto[3,4], Miguel Viveiros[3,5], Pedro V. Baptista[1,*]

[1]CIGMH, Departamento de Ciências da Vida, Faculdade de Ciências e Tecnologia, Universidade Nova de Lisboa, Caparica, Portugal

[2]CENIMAT/I3N, Departamento de Ciência dos Materiais, Faculdade de Ciências e Tecnologia, Universidade Nova de Lisboa, Caparica, Portugal

[3]Grupo de Micobactérias, Unidade Microbiologia Médica, Universidade Nova de Lisboa (IHMT/UNL), Portugal

[4]CREM, Centro de Recursos Microbiológicos, Universidade Nova de Lisboa, Portugal

[5]Centro de Malária e Outras Doenças Tropicais, Instituto de Higiene e Medicina Tropical, Universidade Nova de Lisboa (IHMT/UNL), Portugal

[†] Both authors contributed equally for this work

* Corresponding author: Nanotheranostics@CIGMH, Departamento de Ciências da Vida, Faculdade de Ciências e Tecnologia, Universidade Nova de Lisboa, Campus de Caparica, 2829-516 Caparica, Portugal; Phone/Fax: +351 21 2948530; Email: pmvb@fct.unl.pt

Au-nanoparticles Characterization

Au-nanoparticles were characterized by Transmission Electron Microscopy (TEM) analysis. Ten images were taken in a total of 386 particles analyzed and the mean diameter of ~13.9 nm determined using Carnoy 2.0.

A)

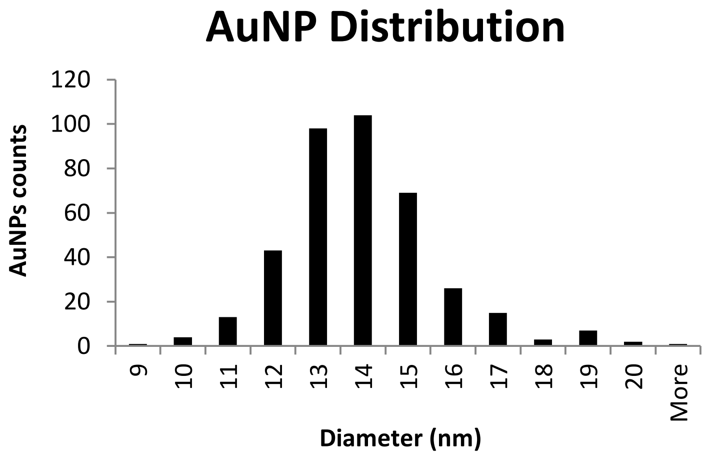

B)

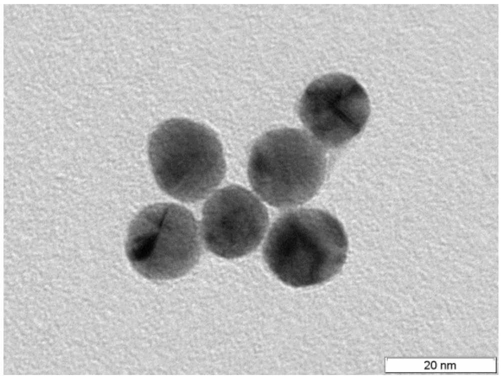

Figure S1. Size and shape of the synthesized AuNPs. (A) histogram of analyzed particles by measured diameter, with a mean diameter of 13.9 nm (± 1.7 nm), (B) TEM picture of spherical nanoparticles with approximate 14 nm diameter size.

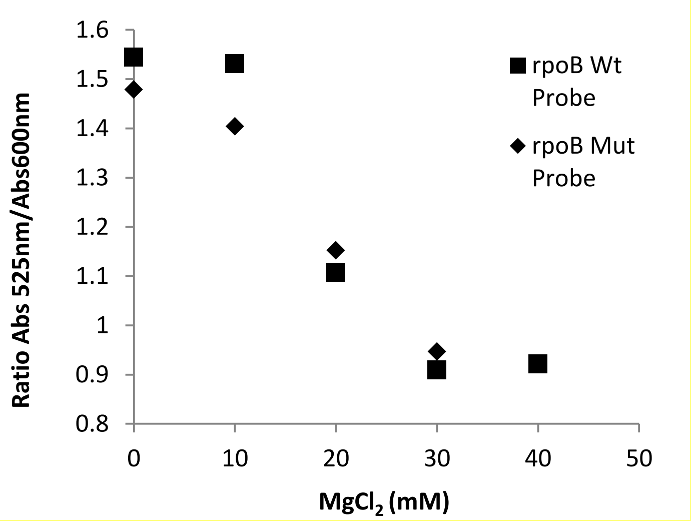

Figure S2. Aggregation profiles of the synthesized Au-nanoprobes. Aggregation measured as the ratio of localized surface plasmon resonance intensity at 525 nm and 600 nm for increasing salt concentrations ($MgCl_2$). The minimum amount of salt required to cause aggregation was determined based on each Au-nanoprobe aggregation profiles. Ratios <1 were considered for full aggregation. Determined concentrations for full aggregation: *rpoB* Wt probe, 30mM; *rpoB* Mut probe 30mM.

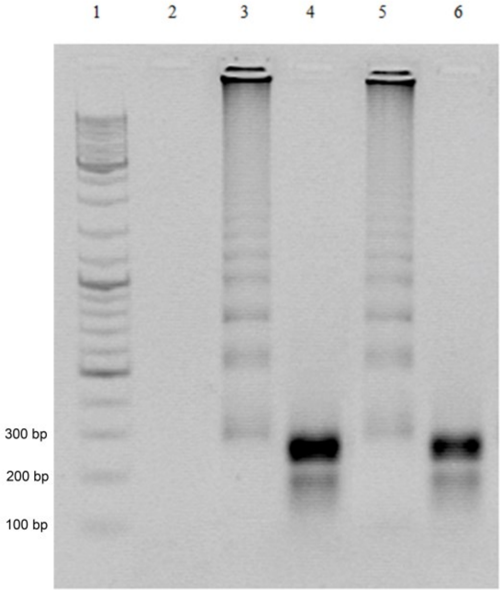

Figure S3. Agarose gel electrophoresis analysis of LAMP products. Electrophoretic analysis in 1% agarose gel of LAMP products (Lane 3 and 5) and digested products with *Apa*I (Lane 4 and 6). Lane 1: Thermo Scientific GeneRuler DNA Ladder Mix (100-10,000 bp)

Table S4. Discrimination of mutations

Sample	Ratio WT/Mut	*rpoB* S531L Genotype
1	1.11	-
2	1.11	-
3	1.08	-
4	1.14	-
5	1.01	-
6	1.09	-
7	0.90	+
8	0.92	+
9	0.93	+
10	0.91	+
11	0.93	+
12	0.93	+

Note: A ratio between spectral data results for each probe was calculated [$WT_{Abs525nm/Abs600nm}$ / $Mut_{Abs525nm/Abs600nm}$]. Values >1 mean higher stability of WT probes, and <1 higher stability of Mut probes. Mutated genotypes (+) and wild type (-)
